# Effects of *Dorema ammoniacum *Gum on Neuronal Epileptiform Activity-Induced by Pentylenetetrazole

**Published:** 2018

**Authors:** Fatemeh Ghasemi, Hanieh Tamadon, Narges Hosseinmardi, Mahyar Janahmadi

**Affiliations:** a *Department of Physiology, Medical School, Shahid Beheshti University of Medical Sciences, Evin, Tehran, Iran. *; b *Neuroscience Research Center, Department of Physiology, Medical School, Shahid Beheshti University of Medical Sciences, Evin, Tehran, Iran.*

**Keywords:** Epileptic activity, Pentylenetetrazole, Dorema ammoniacum, Intracellular recording, Neuronal excitability

## Abstract

Epilepsy is a chronic neurological disease which disrupts the neuronal electrical activity. One-third of patients are resistant to treatment with available antiepileptic agents. The use of herbal medicine for treating several diseases including epilepsy is on the rise. Therefore, further investigation is required to verify the safety and effectiveness of Phytomedicine in treating diseases. The current study is an attempt to elucidate the electrophysiological mechanism of the effect of *Dorema ammoniacum* gum on a cellular model of epilepsy, using intracellular recording method. The gum was applied either after or before pentylenetetrazole, as an epileptic drug, in order to explore the possible therapeutic and preventive effects of gum. Treatment with *D. ammoniacum* gum alone increased the neuronal excitability and when applied before or after treatment with PTZ not only did not prevent or change the electrophysiological changes induced by PTZ but also re-enhanced the induction of hyperexcitability and epileptiform activity through depolarizing membrane potential, increasing the firing frequency and decreasing the AHP amplitude. However, phenobarbital, as a standard anti-epileptic agent, almost reversed the effect of PTZ and preserved the normal firing properties of F1 neurons.

The possible candidate mechanism of the effect of gum on neuronal excitability could be suppressive effects of gum on voltage and/or Ca^2+ ^dependent K^+^ channels currents underlying AHP.

## Introduction

Epilepsy is one of the most common chronic neurological diseases in the world, affecting almost 70 million people (1, 2), in which 40% of patients are considered to be treatment resistant (3). Therefore, new treatment options need to be tested. In fact, some of the most important and successful drugs are derived from natural products (4, 5).

*Dorema ammoniacum *D. Don (Apiaceae) is a medicinal plant whose gum resin has been used as an anthelmintic and for gastrointestinal disorders in Iranian traditional medicine (6). Antibacterial and vasodilatory effects of this herbal plant have also been reported (7, 8). In addition, its extract has also been used as an anticonvulsant drug in Greek folk medicine (7). Recently, Abizadeh and colleagues (2014) have shown that *Dorema ammoniacum *exerts an anticonvulsant protective effect in a rat model of chemical kindling (9). However, its effect at the cellular level has not been investigated to date. In the present study, the mechanism of the effect of *Dorema ammoniacum *gum resin on epileptiform activity and neuronal hyperexcitability induced by PTZ in snail neurons was assessed.

## Experimental

Conventional intracellular recording technique under current clamp condition was performed on F1neuron of *Helix aspersa* (Iranian garden snail). Animals were collected from north of Iran. They were maintained at room temperature (23 ± 2 °C), in a 12-h light/12-h dark cycle, and were fed fresh lettuce ad libitum. All experiments were conducted in accordance with protocols approved by the Shahid Beheshti University of Medical Sciences.

First, the shell of the snail was removed using a bone cutter and a vertical incision was made on the head in order to expose the ganglionic mass. Then, the ganglion was dissected out and transferred into a Sylgard covered chamber and pinned by the nerves and edges of the connective tissue to the bottom of the recording chamber. The superﬁcial layers of the connective tissue surrounding the neurons of ganglion were torn off using forceps without any proteolytic enzymes. F1 neuron was then identiﬁed by location, its size, and colour. The neuronal spontaneous firing activity was recorded in a normal snail Ringer solution containing (in mM): KCl (4), MgCl_2_ (5), CaCl_2_ (10), NaCl (80) HEPES (5), Glucose (10). 

Axoclamp 2B ampliﬁer (Axon Instrument, USA) was used to intracellularly record under current clamp condition from F1 neurons. The reference electrode in all experiments was an Ag/AgCl within an agar bridge (%4 agar in snail Ringer). Microelectrodes were filled with KCl (3M) having resistances of 2-5(ΜΩ).

Neuronal activity was recorded before (control), and after exposure to PTZ (25 mM) and following combination treatment with either *D. ammoniacum* gum and PTZ or phenobarbital and PTZ. Then, recorded data were digitized using a 16-bit A/D converter (ADInstrument, Australia) and stored for further analysis using Peak analysis (ADInstrument), Graph Pad prism, Mini analysis (Synaptosoft Inc.) and SPSS softwares.

The experimental groups were divided into two main groups. In one group, gum was added to the Ringer solution after PTZ application in order to examine the possible therapeutic effect of gum on an epileptic model. The other group, gum was extracellularly applied before induction of epileptiform activity induced by PTZ in order to assess the protective effect of gum. Then, each group was subdivided into several groups. The first group consisted of PTZ + Gum 0.01%, PTZ + Gum 0.1%, PTZ + Gum 0.3% and PTZ + Phenobarbital (600 µΜ) (10). The second group included Gum 0.1% + PTZ, Gum 0.3% + PTZ and Phenobarbital (600 µΜ) + PTZ.

For each experimental group, at least 5 cells with stable resting potential greater than -39 mV were recorded and analyzed. Following 5-10 min of control recording, in protective group, 0.1% and 0.3% concentrations of Gum *ammoniacum* or phenobarbital were applied and recordings were made for 10 min, then PTZ was added in the presence of gum or phenobarbital. In the therapeutic group, first PTZ (25 mM) was applied to the Ringer solution and after 5 min of recording either gum *ammoniacum* (0.01%, 0.1% and 0.3%) (10) or phenobarbital was added to the Ringer solution containing PTZ. 

One-way analysis of variance (ANOVA) followed by Tukey’s test post-hoc was used to examine significant differences between the groups. The results were reported as mean ± SEM. The mean difference was considered significant at the 0.05 level. 

## Results

**Figure 1:**
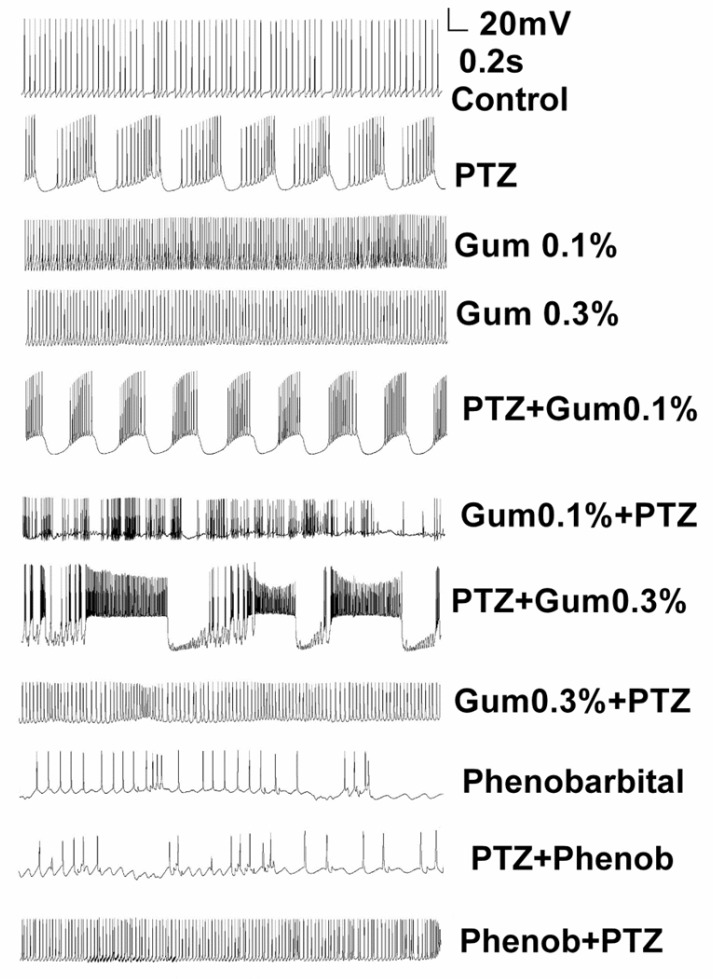
Application of Dorema ammoniacum gum did not reduce or prevent the PTZ-induced epileptiform discharges. Example traces showing spontaneous firing activity for representative neurons in control, after treatment of PTZ alone and following application of PTZ either prior or after exposure to gum

The F1 neuron of *Helix aspersa* exhibited spontaneous rhythmic activity in control condition (Figure 1). Following application of PTZ (25 mM), paroxysmal depolarization shift (PDS), the characteristic of epileptiform activity, and burst activity was induced (Figure 1). Then, in order to evaluate the therapeutic effect of gum *ammoniacum* against epileptiform activity induced by PTZ, three concentrations of gum were added (0.01%, 0.1% and 0.3%) to the Ringer solution containing PTZ. Although the application of gum on PTZ-treated neurons apparently changed the bursting pattern, it did not completely eliminate the epileptiform activity induced by PTZ (Figure 1). Next, alterations in the action potential characteristics were examined under different experimental conditions.

*Effects of PTZ on the electrophysiological properties of F1cells*

**Figure 2.**
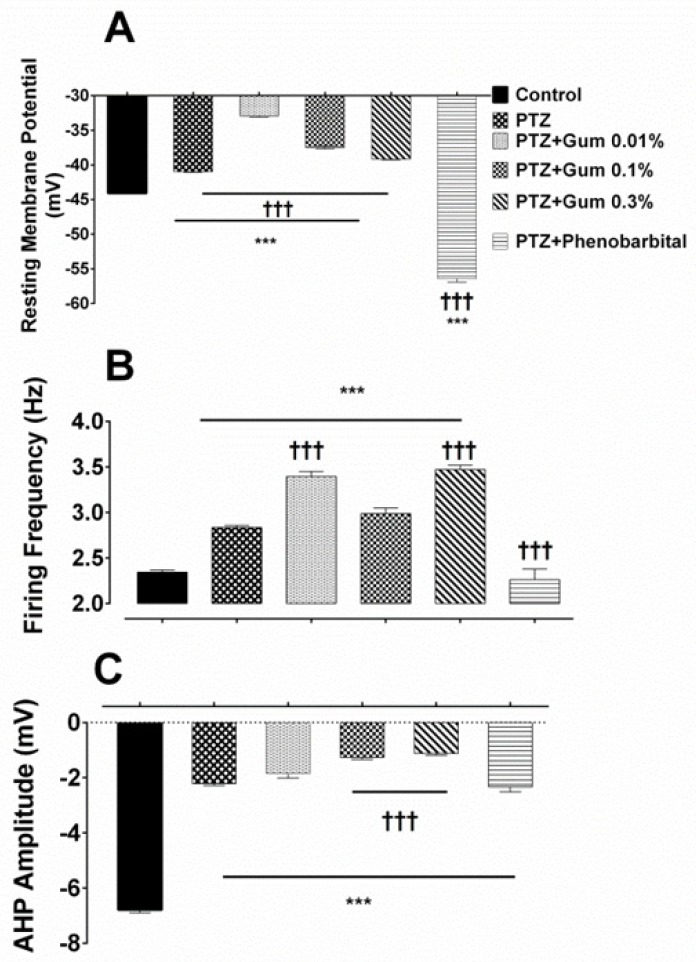
The *ammoniacum* gum did not eliminate the effect of PTZ on electrophysiological properties of F1 neuron. (A) Summary histograms indicating the effect of PTZ alone and in combination with gum on (B) resting membrane potential firing frequency and (C) AHP amplitude. *** and †††*p *< 0.001, represent the significant difference compared to control and PTZ, respectively

Acute exposure to PTZ resulted in a significant depolarization of the resting membrane potential (RMP) (from -44.08 ± 0.11 mV in control *vs* -40.97 ± 0.1 mV after PTZ; *p *≤ 0.001; Figure 2A), and a significant increase in the firing frequency (from 2.34 ± 0.022 Hz in control *vs.* 2.83 ± 0.024 in the presence of PTZ; *p* ≤ 0.001; Figure 2B). It reduced also significantly the AHP amplitude (from -6.78 ± 0.11 mV in control *vs.* -2.21 ± 0.06 mV following PTZ; *p *≤ 0.001; Figure 2C).

**Figure 3:**
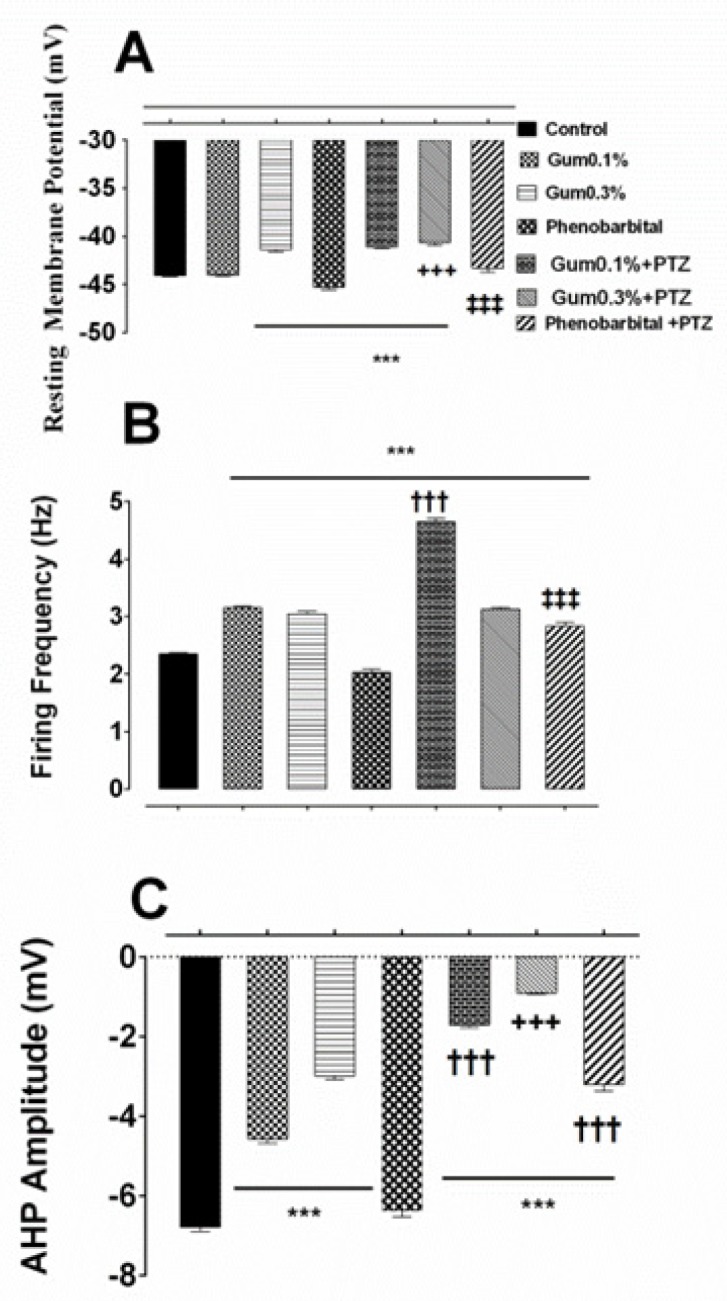
The prophylactic effects of the *ammoniacum* gum on alterations-induced by PTZ in F1 neuron. Effect of pre-treatment of gum on the induction of changes in the (A) resting membrane potential, (B) firing frequency and (C) AHP amplitude of F1 neuron. ***, †††, +++ and ‡‡‡ *p *< 0.001, indicate the significant difference compare to control, gum 0.1%, gum 0.3% and phenobarbital, respectively

*Assessment the possible therapeutic effects of gum ammoniacum or phenobarbital on electrophysiological alterations induced by PTZ*

Bath application of *D. ammoniacum* gum at concentrations of 0.01%, 0.1%, and 0.3% following PTZ exposure, further depolarized the membrane potential which was significantly different when compared with either control or PTZ alone groups (-32.91 ± 0.19 mV, -37.47 ± 0.23 mV and -39.16 ± 0.11 mV in the presence of PTZ plus gum 0.01%, 0.1% and 0.3%, respectively *p* ≤ 0.001; Figure 2A).

Combined application of PTZ plus gum (0.01%, 0.1% or 0.3%) significantly enhanced the neuronal excitability as evidenced by increased firing frequency when compared either to control or PTZ alone (3.393 ± 0.05 Hz, 2.985 ± 0.062 Hz and 3.47 ± 0.04 Hz, following exposure to PTZ plus 0.01%, 0.1% and 0.3%, respective, *p* < 0.001; Figure 2B). 

The amplitude of AHP was also affected, but the reduction in AHP amplitude was significant in the presence of either PTZ + gum 0.1% or PTZ + gum 0.3% containing Ringer solutions (-1.263 ± 0.07 mV and -1.122 ± 0.06 mV, *p* ≤ 0.001; Figure 2C).

Application of phenobarbital (600 µM), a standard antiepileptic drug, in combination with PTZ shifted significantly the membrane potential toward a hyperpolarized potential (-56.41 ± 0.5 mV, *p* ≤ 0.001 compared to control and PTZ alone; Figure 2A), significantly reduced the neuronal firing frequency (2.262 ± 0.11 Hz, *p* ≤ 0.001) (Figure 2B) and increased the amplitude of AHP (-2.33 ± 0.18 mV; *p *= 0.99; Figure 2C) compared to PTZ alone, but still was significantly lower than that recorded in control condition (*p* < 0.001). 

*Changes in the electrophysiological properties of F1 neurons in the presence of PTZ following Pre-application of gum ammoniacum or phenobarbital: Assessing the protective effect of gum ammoniacum*

Next, the possible protective effect of gum *ammoniacum* against induction of epileptiform activity by PTZ was examined. Therefore, after perfusion of Ringer solution containing higher concentrations of gum (0.1% or 0.3%), PTZ (25 mM) was added to the same Ringer solution then electrophysiological responses were recorded. 

Statistical analysis of electrical responses showed that when 0.1% or 0.3% concentrations of gum *ammoniacum* were applied following control recording of spontaneous activity, the RMP was depolarized and the effect was more prominent in the presence of gum 0.3% (-41.37 ± 0.2 mV for gum 0.3%, *p* ≤ 0.001; Figure 3A). Exposure to PTZ following gum *ammoniacum* (0.1% or 0.3%), depolarized the RMP and the effect was only significant for the gum 0.1% + PTZ group, compared to gum treatment alone (-41.10 ± 0.14 mV, *p* ≤ 0.001; Figure 3A). 

Firing frequency of action potential significantly increased following application of gum *ammoniacum* (3.155 ± 0.02 Hz and 3.04 ± 0.03 Hz, in the presence of 0.1% or 0.3%, respectively, *p* < 0.001 compared to control group; Figure 3B), and when PTZ was added, further increase in the firing frequency was observed which was significant for gum 0.1% + PTZ group (4.64 ± 0.061 Hz, *p* < 0.001; Figure 3B).

Application of gum alone at concentration of either 0.1% or 0.3% resulted in a significant decrease in the AHP amplitude when compared to control values (-4.567 ± 0.112 mV, -2.989 ± 0.09 mV, following application of gum 0.1% and 0.3%, respectively; *p *≤ 0.001; Figure 3C). However, when neurons pretreated with gum (0.1% or 0.3%) were exposed to PTZ a significant decrease in AHP (-1.714 ± 0.06 mV and -0.908 ± 0.03 mV, *p* ≤ 0.001; Figure 3C) was evidenced when compared to the effect of gum 0.1% and 0.3% alone.

Next, the preventive effect of phenobarbital, which is considered as a standard anticonvulsant therapy, on the electrical properties of the F1 neuron was investigated. Application of phenobarbital alone to the bathing solution led to a significant shift in membrane potential toward hyperpolarized potential (-45.30 ± 0.25 mV; *p* ≤ 0.001; Figure 3A). Exposure of phenobarbital pretreated neurons to PTZ shifted the membrane potential towards less negative potential and restored the membrane potential almost to the control level (-43.29 ± 0.43 mV; Figure 3A).

Treatment with phenobarbital alone decreased also significantly the firing frequency (2.02 ± 0.05 Hz, *p* ≤ 0.001; Figure 3B), but application of PTZ following phenobarbital exposure, led to a significant increase in the firing frequency when compared to the control or the gum (0.1% or 0.3%) groups (2.82 ± 0.072 Hz, *p *≤ 0.001; Figure 3B). 

Finally, Phenobarbital itself produced a significant change in the amplitude of AHP compared to either control or gum applied groups (-6.375 ± 0.15 mV, *p* ≤ 0.001; Figure 3C), but when applied in combination with PTZ caused a significant decrease in AHP amplitude (-3.204 ± 0.162 mV, *p* ≤ 0.001; Figure 3C).

## Discussion

Epilepsy affects more than 70 million people in the world and is one of the oldest known and most common neurological disorders which disrupt neuronal function (1, 2). However, there is no available effective radical preventive and/or therapeutic therapy. Medicinal plants for having a wide variety of phytochemical compounds are used in the treatment of diseases, including epilepsy. However, understanding the cellular mechanisms underlying the action of herbal medicine at the cellular level may help to elucidate the safety and effectiveness of them. Therefore, in the current study, we aimed to investigate the cellular effects of ammonium gum on neuronal excitability in an epileptic model.

*Dorema ammoniacum *D. Don. (Apiaceae) is one of the most important endemic medicinal plants in some of the Asian countries including Iran (11). It produces a medicinal gum resin commonly known as *ammoniacum* gum. In Iranian folk medicine, its gum resin has been considered beneficial in the treatment of spastic pains, intestinal parasitic infections, skin inflammation, stimulant, antimicrobial, and vasodilator (12-14). The gum also is used in food industry, confectionery products (14); therefore, investigating the effect of gum on neuronal activity is essential. 

Findings of the present study indicated that in application of gum alone caused an increase in firing frequency and decreased AHP amplitude. Pretreatment with gum did not eliminate the ability of PTZ to depolarize the membrane potential, to induce hyperexcitability, and to reduce AHP amplitude. 

However, when phenobarbital was used as an antiepileptic drug and then its effect on excitability was compared with those of gum, we found that both in therapeutic and preventive groups, phenobarbital reduced neuronal excitability. 

On the other hand, in the therapeutic group in which gum was applied after induction of epileptiform activity did not only reduce the neuronal excitability induced by PTZ, but also resulted further increase in the neuronal epileptiform activity. This was associated with further membrane depolarization, higher firing frequency, and lower AHP amplitude.

There is so far, no report describing the effect of *ammoniacum* gum at the cellular level and the exact mechanism by which the gum affect the neuronal excitability is not known; however, there are several possible explanations which have been deduced from the current clamp results. First, exposure to gum alone significantly decreased the AHP amplitude which thereby decreased neuronal excitability. Consistent with this finding, there are many evidence suggesting that AHP regulates the neuronal firing rate (15-18).

Here, we also showed that both therapeutic and preventive treatments of gum did not produce a clear antiepileptic effect against epileptiform induction by PTZ, while phenobarbital, as a standard antiepileptic agent, showed inhibitory effect both in therapeutic and prophylactic groups. Our results are in contrast to what Abizadeh *et al.* (2014) and Motevalian *et al.* (2017) observed behaviourally in an *in-vivo* model of epilepsy (9, 19). They reported the *in-vivo* anti-epileptic activity of *Dorema ammoniacum* gum in a rat and mice model of epilepsy induced by PTZ, respectively. Based on these *in-vivo* studies, gum appeared to have antiepileptogenic activity; however, in the present account not only the gum was a lack of antiepileptic action when applied before and after application of PTZ, but also its application alone was associated with neuronal hyperexcitability. 

The discrepancy between *in-vivo* and *in-vitro* antiepileptic activity of the gum could be due to the animal species (rat *vs.* snail) or to the different route of gum application and possibly the dosage. In both works previously published by Abizadeh *et al.* and Motevalian *et al.*, *Dorema ammoniacum* gum was intraperitoneally injected. In systemic injection of *Dorema ammoniacum *gum, some of the compounds in the gum that may exert excitatory action probably have been metabolized in live, which is the principal site of drug metabolism. Therefore, the excitatory fraction (s) of the gum has been greatly reduced or inactivated. Whereas, here, the direct action of the whole gum was assessed using intracellular recording technique. An excitatory compound from gum *ammoniacum* with acetylcholinesterase (AchE) inhibitory activity has been isolated and characterized by Adhami and colleagues (20), which may provoke seizure (21, 22). 

Overall, it can be concluded that *ammoniacum* gum in therapeutic usage potentiates the PTZ-induced hyperexcitation by reducing the amplitude of AHP and depolarizing the neuron which suggests the possible damping effect of gum on Ca^2+ ^and/or voltage -dependent K^+^ channels; although this needs to be further explored by voltage clamp method. In addition, these findings indicate that a certain caution should be taken into account when *ammoniacum* gum is used.
